# Use of GLP-1 Receptor Agonists and Occurrence of Thyroid Disorders: a Meta-Analysis of Randomized Controlled Trials

**DOI:** 10.3389/fendo.2022.927859

**Published:** 2022-07-11

**Authors:** Weiting Hu, Rui Song, Rui Cheng, Caihong Liu, Rui Guo, Wei Tang, Jie Zhang, Qian Zhao, Xing Li, Jing Liu

**Affiliations:** ^1^The Second Clinical Medical College, Shanxi Medical University, Taiyuan, China; ^2^Department of Endocrinology, Second Hospital of Shanxi Medical University, Taiyuan, China; ^3^Department of Physiology, Shanxi Medical University, Taiyuan, China

**Keywords:** GLP-1 receptor agonists, thyroid disorders, thyroid cancer, meta-analysis, randomized controlled trials

## Abstract

The association between glucagon-like peptide-1 (GLP-1) receptor agonists and the risk of various kinds of thyroid disorders remains uncertain. We aimed to evaluate the relationship between the use of GLP-1 receptor agonists and the occurrence of 6 kinds of thyroid disorders. We searched PubMed (MEDLINE), EMBASE, the Cochrane Central Register of Controlled Trials (CENTRAL) and Web of Science from database inception to 31 October 2021 to identify eligible randomized controlled trials (RCTs). We performed meta-analysis using a random-effects model to calculate risk ratios (RRs) and 95% confidence intervals (CIs). A total of 45 trials were included in the meta-analysis. Compared with placebo or other interventions, GLP-1 receptor agonists’ use showed an association with an increased risk of overall thyroid disorders (RR 1.28, 95% CI 1.03-1.60). However, GLP-1 receptor agonists had no significant effects on the occurrence of thyroid cancer (RR 1.30, 95% CI 0.86-1.97), hyperthyroidism (RR 1.19, 95% CI 0.61-2.35), hypothyroidism (RR 1.22, 95% CI 0.80-1.87), thyroiditis (RR 1.83, 95% CI 0.51-6.57), thyroid mass (RR 1.17, 95% CI 0.43-3.20), and goiter (RR 1.17, 95% CI 0.74-1.86). Subgroup analyses and meta-regression analyses showed that underlying diseases, type of control, and trial durations were not related to the effect of GLP-1 receptor agonists on overall thyroid disorders (all P _subgroup_ > 0.05). In conclusion, GLP-1 receptor agonists did not increase or decrease the risk of thyroid cancer, hyperthyroidism, hypothyroidism, thyroiditis, thyroid mass and goiter. However, due to the low incidence of these diseases, these findings need to be examined further.

**Systematic Review Registration:**

PROSPERO https://www.crd.york.ac.uk/prospero/, identifier: CRD42021289121.

## Introduction

Thyroid diseases are common in some metabolic disorders, such as diabetes mellitus (DM) and obesity. Thyroid dysfunction (TD) and DM are closely linked. A high prevalence of TD has been reported among both type 1 DM (T1DM) and type 2 DM (T2DM) patients (1, 2). Although the mechanism is unknown, epidemiological studies have indicated that obesity and T2DM are associated with increased risks of several cancers, including thyroid cancer (3–5). Furthermore, insulin resistance and hyperinsulinemia can lead to goiter, proliferation of thyroid tissues, and an increased incidence of nodular thyroid disease (6). In addition to the effects of the disease itself, some antidiabetic drugs can impact the hypothalamic–pituitary–thyroid (HPT) axis and thyroid function. For example, multiple studies have demonstrated that metformin can inhibit the growth of thyroid cells and different types of thyroid cancer cells, and metformin therapy has been associated with a decrease in the levels of serum thyroid-stimulating hormone (TSH) (7). Thiazolidinediones can induce thyroid-associated ophthalmopathy (8, 9). Recently, the relationship between glucagon-like peptide-1 (GLP-1) receptor agonists and thyroid cancer has attracted attention, but there is still controversy.

GLP-1 is an amino acid peptide hormone secreted by L cells of the gastrointestinal mucosa that promotes insulin secretion, suppresses glucagon secretion, and delays gastric emptying (10). Rodent studies have shown that the GLP-1 receptor agonist liraglutide can activate the GLP-1 receptor on thyroid C cells, leading to the release of calcitonin with a dose-dependent effect on the pathology of C cells (11). Some animal models have proven that exenatide or liraglutide treatment is related to the abnormal appearance of thyroid C cells, with gradual development of hyperplasia and adenomas (12, 13). Moreover, a study found that patients treated with exenatide had an increased risk of thyroid cancer by examining the US Food and Drug Administration’s database of reported adverse events (14). However, the results of A Long Term Evaluation (LEADER) trial that followed for 3.5-5 years showed no effect of GLP-1 receptor activation on human serum calcitonin levels, C-cell proliferation or C-cell malignancy (15). Nevertheless, GLP-1 receptor agonists are not recommended in patients with a personal or family history of medullary thyroid cancer or type 2 multiple endocrine neoplasia.

GLP-1 receptor agonists, a new type of antidiabetic drug for treating T2DM in recent years, with additional benefits of weight loss and blood pressure reduction (16). Although many large randomized controlled trials (RCTs) of GLP-1 receptor agonists have identified the obvious benefits of GLP-1 receptor agonists on cardiovascular and renal outcomes in patients with DM or obesity (17–20), the association between GLP-1 receptor agonists and various thyroid disorders remains controversial. In addition, considering that thyroid disorders are common in some metabolic diseases such as DM and obesity, we conducted this study. Thus, by comparing GLP-1 receptor agonists with placebo or other antidiabetic drugs, we conducted a meta-analysis of all available RCT data to evaluate the relationship between the use of GLP-1 receptor agonists and the occurrence of various kinds of thyroid disorders.

## Methods

### Data Sources and Searches

We searched PubMed (MEDLINE), EMBASE, Cochrane Central Register of Controlled Trials (CENTRAL) and Web of Science from database inception to 31 October 2021 to identify eligible RCTs without restriction of language or publication period. The search terms used were “glucagon-like peptide 1 receptor agonist”, “exenatide”, “liraglutide”, “dulaglutide”, “lixisenatide”, “semaglutide”, “albiglutide”, “taspoglutide”, “loxenatide”, “diabetes mellitus”, “obesity” and “randomized controlled trial”. In addition, we manually scanned the ClinicalTrials.gov web and reference lists from established trials and review articles.

### Study Selection

The trials we included met the following criteria: (1) RCTs that compared GLP-1 receptor agonist with a placebo or active control (other antidiabetic drugs or insulin), (2) patients with type 2 diabetes, type 1 diabetes, prediabetes, overweight or obesity, (3) with durations of at least 24 weeks, and (4) reported the occurrence of at least one case of various thyroid disorders as adverse events. We excluded duplicate reports, conference abstracts, letters, case reports, editorials, articles without treatment-emergent adverse events, and animal experimental studies.

### Data Extraction and Quality Assessment

Two investigators (Hu and Song) independently extracted the following data by reviewing the full text of each study: first author, year of publication, Clinical Trial Registration Number (NCT ID), trial duration, patient characteristics, sample size, intervention (type of GLP-1 receptor agonist), comparators, and outcomes of interest. Any discrepancies were resolved by consensus or by the third reviewer (Chen). The primary outcome was the incidence of overall thyroid disorders, and the secondary outcomes included the incidence of goiter, hyperthyroidism, hypothyroidism, thyroiditis, thyroid mass, and thyroid cancer. When multiple reports from the same population were retrieved, the most complete or recently reported data were used. If thyroid-related events were not reported in publication, these data were extracted from the ‘Serious Adverse Events’ portion of ClinicalTrials.gov.

The quality of each included RCT was assessed by the Cochrane Risk-of-Bias Tool 1.0. The Jadad scale was also used to quantify the study quality. Two authors assessed the risk of bias for each study through five aspects: random sequence generation, allocation concealment, blinding, incomplete outcome data and selective reporting.

### Statistical Analysis

Dichotomous outcomes were analyzed by risk ratios (RRs) and 95% confidence intervals (CIs) using the DerSimonian and Laird random-effects model. We assessed heterogeneity between the included studies using the I² statistic, where I^2^ values of 25%, 50%, and 75% indicated low, medium, and high heterogeneity, respectively. Subgroup analyses were conducted according to the type of underlying diseases, type of control, and trial duration. Between-subgroup heterogeneity was assessed by χ^2^ tests and meta-regression. All of the above analyses were performed using Stata software 13.0 (Stata Corp). A p value < 0.05 was considered statistically significant.

## Result

### Study Search and Study Characteristics

**Figure 1:**
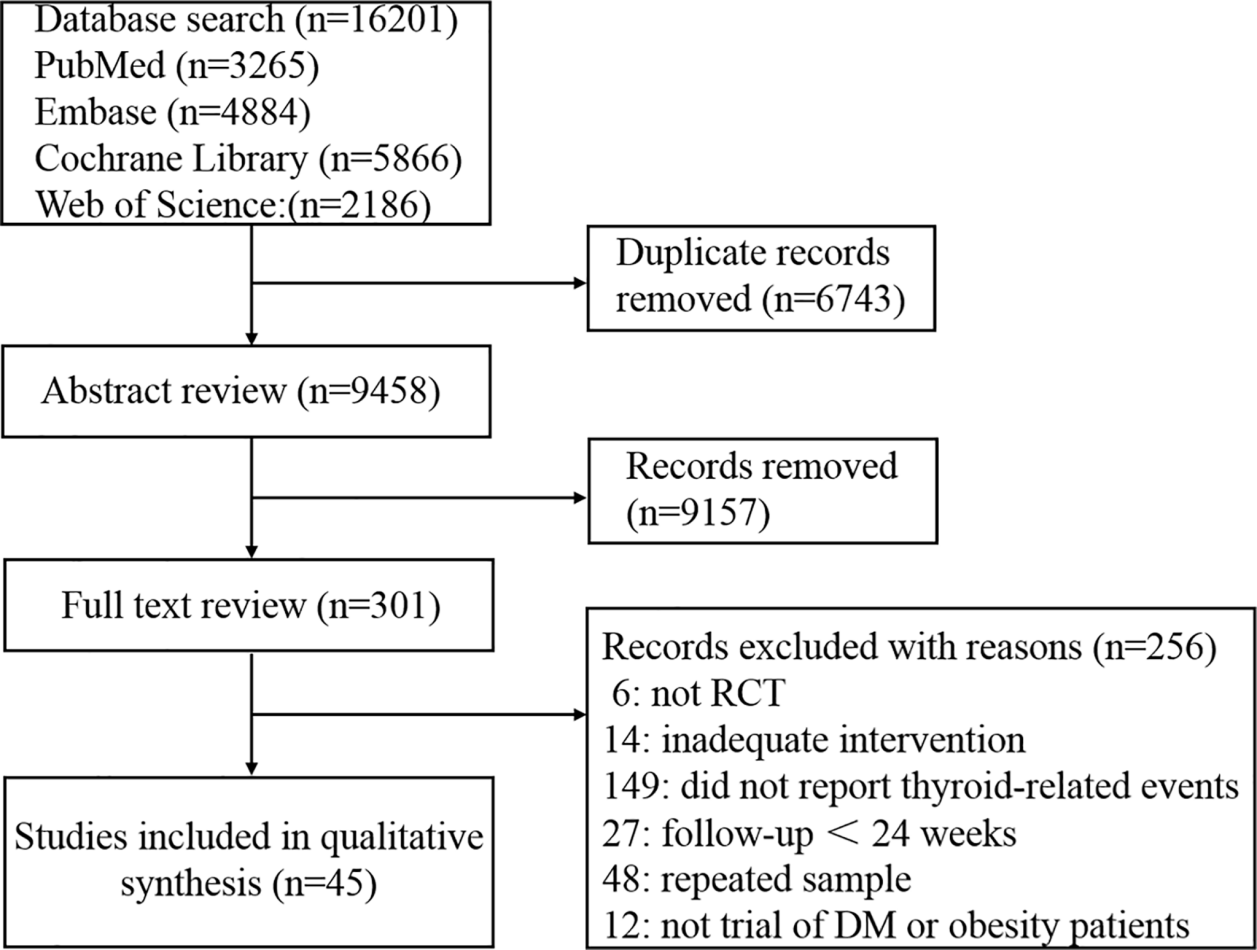
Summary of trial selection.

A total of 16,201 records were identified by retrieving the aforementioned databases. Excluding duplicates and reviewing titles and abstracts, 301 studies were read the full text. After retrieving the full text and searching on ClinicalTrials.gov, the final analysis included 45 RCTs reported in 45 publications with 94063 participants (17–61). Although the data from the two articles were presented together on ClinicalTrials.gov (62), due to the differences in population characteristics and follow-up time, we considered them separately and regarded them as two independent trials (24, 25). The search and selection process is summarized in **Figure 1**. The characteristics of these included studies are detailed in **Table 1** and **Table S1**. Across the 45 trials, trial duration ranged from 26 to 360 weeks. Of all the participants, 29,348 (55.8%) were men in the experimental group, and 24121 (58.2%) were men in the control group. The mean age of study participants ranged from 41.6 to 66.2 years old in experimental groups and 41.4 to 66.2 years old in control groups. Mean patient body mass index (BMI) ranged from 24.5 to 39.3 kg/m2 in experimental groups and 24.4 to 39.0 kg/m2 in control groups.

**Table 1:** Baseline characteristics of included studies.

Study	Clinical Trial Registration Number	Trial Duration (week)	Interventions		Events/Patients (N)		Age (years)		Man (N, %)		BMI (kg/m^2^)		Jadad score
			Experimental	Control	Experimental	Control	Experimental	Control	Experimental	Control	Experimental	Control	
Unger et al., 2022 (21)	NCT02730377	105	Liraglutide	OAD	1/996	0/995	57.6 (11.0)	57.1 (10.7)	520 (52.2)	524(52.7)	33.2 (7.2)	33.7(7.6)	2
Garveyet al 2020 (22)	NCT02963922	60	Liraglutide	Placebo	1/198	0/198	55.9 (11.3)	57.6 (10.4)	90 (45.5)	99 (50.0)	35.9 (6.5)	35.3(5.8)	4
Wadden et al., 2020 (23)	NCT02963935	60	Liraglutide	Placebo	1/142	0/140	45.4 (11.6)	49.0 (11.2)	23 (16.2)	24 (17.1)	39.3 (6.8)	38.7(7.2)	4
le et al., 2017 (24)	NCT01272219	172	Liraglutide	Placebo	3/1505	3/749	47.5 (11.7)	47.3 (11.8)	364 (24.0)	176 (23.0)	38.8 (6.4)	39.0(6.3)	4
Pi-Sunyer et al., 2015 (25)	NCT01272219	68	Liraglutide	Placebo	1/959	0/487	41.6 (11.7)	41.5 (11.5)	158 (16.5)	97 (19.9)	37.5 (6.2)	37.4(6.2)	4
Zang et al., 2016 (26)	NCT02008682	26	Liraglutide	Sitagliptin	0/183	1/184	51.7 (10.7)	51.4 (11.0)	102 (55.7)	117 (63.6)	27.3 (3.4)	27.2(4.0)	2
Ahrén et al., 2016 (27)	NCT02098395	26	Liraglutide	Placebo	2/625	0/206	43.3	42.7	288 (46.1)	94 (45.6)	28.9	28.9	4
Mathieu et al., 2016 (28)	NCT01836523	52	Liraglutide	Placebo	0/1042	1/347	43.7	43.4	496 (47.6)	167(48.1)	29.4	29.8	4
Marso et al., 2016 (20)	NCT01179048	240	Liraglutide	Placebo	77/4668	54/4672	64.2 (7.2)	64.4 (7.2)	3011 (64.5)	2992 (64.0)	32.5 (6.3)	32.5(6.3)	4
Davies et al., 2015 (29)	NCT01272232	68	Liraglutide	Placebo	1/634	1/212	55.0	54.7	328 (51.7)	97 (45.8)	37.1	37.4	4
Gough et al., 2014 (30)	NCT01336023	52	Liraglutide IDegLira	Degludec	2/414 0/833	0/413	55.0 (10.2) 55.1 (9.9)	54.9 (9.7)	208 (50.2) 435 (52.2)	200(48.4)	31.3 (4.8) 31.2 (5.2)	31.2(5.3)	3
Wadden et al., 2013 (31)	NCT00781937	56	Liraglutide	Placebo	3/212	0/210	45.9 (11.9)	46.5 (11.0)	34 (16.0)	45 (21.4)	38.2 (6.2)	37.5(6.2)	4
Seino et al., 2010 (32)	NCT00393718	52	Liraglutide	Glibenclamide	1/268	0/132	58.2 (10.4)	58.5 (10.4)	183 (68.3)	86 (65.2)	24.5 (3.7)	24.4(3.8)	4
Pratley et al., 2010 (33)	NCT00700817	78	Liraglutide	Sitagliptin	1/446	0/219	55.5	55.0	232 (52.0)	120(55.0)	32.9	32.6	2
Nauck et al., 2009 (34)	NCT00318461	104	Liraglutide	Glibenclamide Placebo	6/724	2/242 0/121	56.7	57.3 56.0	422 (58.3)	139(57.4) 72 (59.5)	30.8	31.2 31.6	4
Garber et al., 2009 (35)	NCT00294723	104	Liraglutide	Glibenclamide	6/498	0/248	52.9	53.4	238 (47.8)	133(53.6)	33.0	33.2	3
Hernandez et al., 2018 (36)	NCT02465515	130	Albiglutide	Placebo	0/4731	1/4732	64.1 (8.7)	64.2 (8.7)	3304 (70.0)	3265(69.0)	32.3 (5.9)	32.3(5.9)	5
Home et al., 2015 (37)	NCT00839527	52	Albiglutide	Pioglitazone Placebo	5/271	9/277 2/115	54.5 (9.5)	55.7 (9.4) 55.7 (9.6)	135 (49.8)	148(53.4) 70 (60.9)	32.4 (5.5)	32.2(5.7) 31.8(4.9)	3
Ahrén et al., 2014 (38)	NCT00838903	164	Albiglutide	Sitagliptin Glibenclamide Placebo	1/302	2/302 0/307 0/101	54.3 (10.1)	54.3 (9.8) 54.4 (10.0) 56.1(10.0)	135 (44.7)	139(46.0) 158(51.5) 50 (49.5)	32.7 (5.6)	32.5(5.4) 32.5(5.5) 32.8(5.4)	2
Leiter et al., 2014 (19)	NCT01098539	60	Albiglutide	Sitagliptin	1/249	0/246	63.2 (8.4)	63.5 (9.0)	136 (54.6)	130(52.8)	30.4 (5.5)	30.4(5.8)	4
Holman et al., 2017 (18)	NCT01144338	360	Exenatide	Placebo	23/7356	16/7396	61.8 (9.4)	61.9 (9.4)	4562 (62)	4587(62)	31.8	31.7	5
Gallwitz et al., 2012 (39)	NCT00359762	216	Exenatide	Glimepiride	0/490	4/487	56.0 (10.0)	56.0 (9.1)	272 (55.5)	252 (51.7)	32.6 (4.2)	32.3(3.9)	2
Bergenstal et al., 2010 (40)	NCT00637273	26	Exenatide	Sitagliptin Pioglitazone	0/160	1/166 0/165	52.4 (10.4)	52.2(10.5) 53.0 (9.9)	89 (55.6)	86 (51.8) 79 (47.9)	32.0 (5.0)	32.0(5.0) 32.0(6.0)	3
Wang et al., 2019 (41)	NCT01648582	56	Dulaglutide	Glargine	8/505	2/250	54.8	55.4	278 (55.0)	139(55.6)	26.8	26.7	2
Gerstein et al., 2019 (42)	NCT01394952	336	Dulaglutide	Placebo	26/4949	14/4952	66.2 (6.5)	66.2 (6.5)	2643 (53·4)	2669 (53·9)	32.3 (5.7)	32.3(5.8)	5
Chen et al., 2018 (43)	NCT01644500	26	Dulaglutide	Glimepiride	2/478	0/242	53.2	52.0	261 (54.6)	130 (53.7)	26.0	25.7	4
Weinstock et al., 2015 (44)	NCT00734474	104	Dulaglutide	Sitagliptin Placebo	3/606	0/315 0/177	54.0	54.0 55.0	280 (46.2)	151(48.0) 90 (51.0)	31.0	31.0 31.0	5
Giorgino et al., 2015 (45)	NCT01075282	78	Dulaglutide	Glargine	1/545	0/262	56.5	57.0	280 (51.4)	134(51.0)	31.5	32.0	2
Rosenstock et al., 2016 (46)	NCT02058147	30	Lixisenatide iGlarLixi	Glargine	0/469 0/234	1/467	58.7 (8.7) 58.2 (9.5)	58.3(9.4)	133 (56.8) 222 (47.3)	237(50.7)	32.0 (4.4) 31.6 (4.4)	31.7(4.5)	2
Pfeffer et al., 2015 (47)	NCT01147250	225	Lixisenatide	Placebo	2/3034	3/3034	59.9 (9.7)	60.6(9.6)	2111 (69.6)	2096(69.1)	30.1 (5.6)	30.2(5.8)	5
Bolli et al., 2014 (48)	NCT00763451	112	Lixisenatide	Placebo	2/322	0/160	55.0	58.2	143 (44.4)	72 (45.0)	32.6	32.4	5
Ahrén et al., 2013 (49)	NCT00712673	76	Lixisenatide	Placebo	1/510	1/170	54.7	55.0	212 (41.6)	81 (47.6)	32.9	33.1	4
Riddle et al., 2013 (50)	NCT00715624	125	Lixisenatide	Placebo	1/328	0/167	57.4 (9.5)	56.9(9.8)	146 (44.5)	82 (49.1)	31.9 (6.2)	32.6(6.3)	5
Wilding et al., 2021 (51)	NCT03548935	75	Semaglutide	Placebo	1/1306	0/655	46.0 (13.0)	47.0 (12.0)	351 (26.9)	157(24.0)	37.8 (6.7)	38.0(6.5)	4
Wadden et al., 2021 (52)	NCT03611582	75	Semaglutide	Placebo	1/407	0/204	46.0 (13.0)	46.0 (13.0)	92 (22.6)	24 (11.8)	38.1 (6.7)	37.8(6.9)	5
Yamada et al., 2020 (53)	NCT03018028	57	Semaglutide Liraglutide	Placebo	1/146 0/48	0/49	59.7 59.0	59.0	112 (76.7) 39 (81.3)	40 (81.6)	25.8 26.9	25.1	5
Husain et al., 2019 (54)	NCT02692716	87	Semaglutide	Placebo	2/1591	2/1592	66.0 (7.0)	66.0(7.0)	1084 (68.1)	1092(68.6)	32.3 (6.6)	32.3(6.4)	5
Rosenstock et al., 2019 (55)	NCT02607865	83	Semaglutide	Sitagliptin	0/1396	1/467	58.0	58.0	746 (53.4)	238(51.0)	32.5	32.5	3
Pratley et al., 2019 (56)	NCT02863419	57	Semaglutide Liraglutide	Placebo	2/285 1/284	0/142	56.0 (10.0) 56.0 (10.0)	57.0(10.0)	147 (51.6) 149 (52.5)	74 (52.1)	32.5 (5.9) 33.4 (6.7)	32.9(6.1)	4
Aroda et al., 2019 (57)	NCT02906930	31	Semaglutide	Placebo	2/525	0/178	55.0	54.0	268 (51.0)	89 (50.0)	31.7	32.2	3
O’Neil et al., 2018 (58)	NCT02453711	59	Semaglutide Liraglutide	Placebo	0/718 0/103	1/136	46.3 49.0	46.0	254 (35.4) 36 (35.0)	48 (35.0)	30.0 30.4	30.7	3
Ahrén et al., 2017 (59)	NCT01930188	56	Semaglutide	Sitagliptin	3/818	0/407	55.4	54.6	412 (50.3)	208(51.1)	32.5	32.5	4
Aroda et al., 2017 (60)	NCT02128932	36	Semaglutide	Glargine	0/722	1/360	56.6	56.2	379 (52.5)	195 (54)	33.1	33.0	3
Marso et al., 2016 (17)	NCT01720446	109	Semaglutide	Placebo	4/1648	6/1649	64.7	64.6	1013 (61.5)	989(60.0)	–	–	4
Gerstein et al., 2021 (61)	NCT03496298	126	Efpeglenatide	Placebo	5/2717	0/1359	64.7	64.4	1792 (66.0)	940(69.2)	32.9	32.4	5

OAD, oral antidiabetic drugs; IDegLira, insulin degludec/liraglutide; IGlarLixi, insulin glargine/lixisenatide Fixed Ratio Combination.

### Risk of Bias Evaluation

The studies included in this analysis provide information about random sequence generation, allocation concealment, participant blindness, personnel, outcome evaluation and selective reporting. **Figure S1** reports the risk details of deviation assessment. (**Figure S1** in **Appendix**) 29 trials had a Jadad scale of 4 or 5, and others were scored ≤3.

### Incidence of Thyroid Disorders With All GLP-1 Receptor Agonists

**Figure 2:**
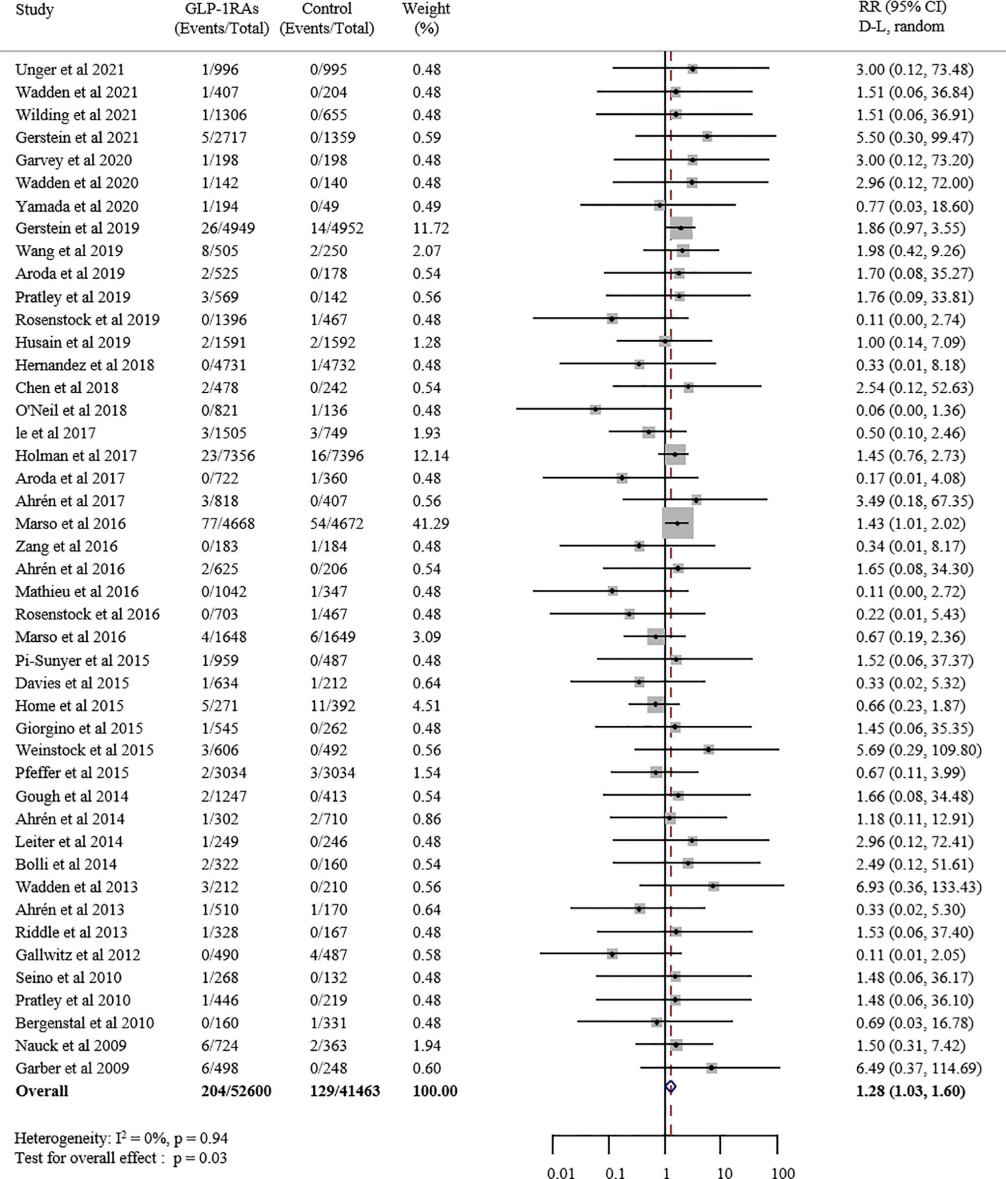
Forest plot of GLP-1 receptor agonists versus comparators on risk of overall thyroid disorders. GLP-1RAs, GLP-1 receptor agonists; RR, risk ratios; CI, confidence interval.

As is shown in **Figure 2**, this meta-analysis included 52600 patients in the GLP-1 receptor agonist group and 41463 patients in the control group. The event rate in the GLP-1 receptor agonist group (0.39%) was higher than in the control group (0.31%). Compared with placebo or other interventions, GLP-1 receptor agonist increased the risk of overall thyroid disorders by 28% (RR 1.28, 95% CI 1.03-1.60; p = 0.027), with no statistically significant between-study heterogeneity (I^2^ = 0.0%). The funnel plot for this analysis indicated no significant publication bias (**Figure S2**).

GLP-1 receptor agonists versus placebo or other interventions had no significant effects on the occurrence of thyroid cancer (RR 1.30, 95% CI 0.86-1.97, p = 0.212; I^2^ = 0.0%; **Figure S3**), hyperthyroidism (RR 1.19, 95% CI 0.61-2.35, p = 0.608; I^2^ = 0.0%; **Figure S4**), hypothyroidism (RR 1.22, 95% CI 0.80-1.87, p = 0.359; I^2^ = 0.0%; **Figure S5**), thyroiditis (RR 1.83, 95% CI 0.51-6.57, p = 0.353; I^2^ = 0.0%; **Figure S6**), thyroid mass (RR 1.17, 95% CI 0.43-3.20, p = 0.759; I^2^ = 0.0%; **Figure S7**), and goiter (RR 1.17, 95% CI 0.74-1.86, p = 0.503; I^2^ = 0.0%; **Figure S8**).

### Incidence of Thyroid Disorders With Different GLP-1 Receptor Agonists

**Figure 3:**
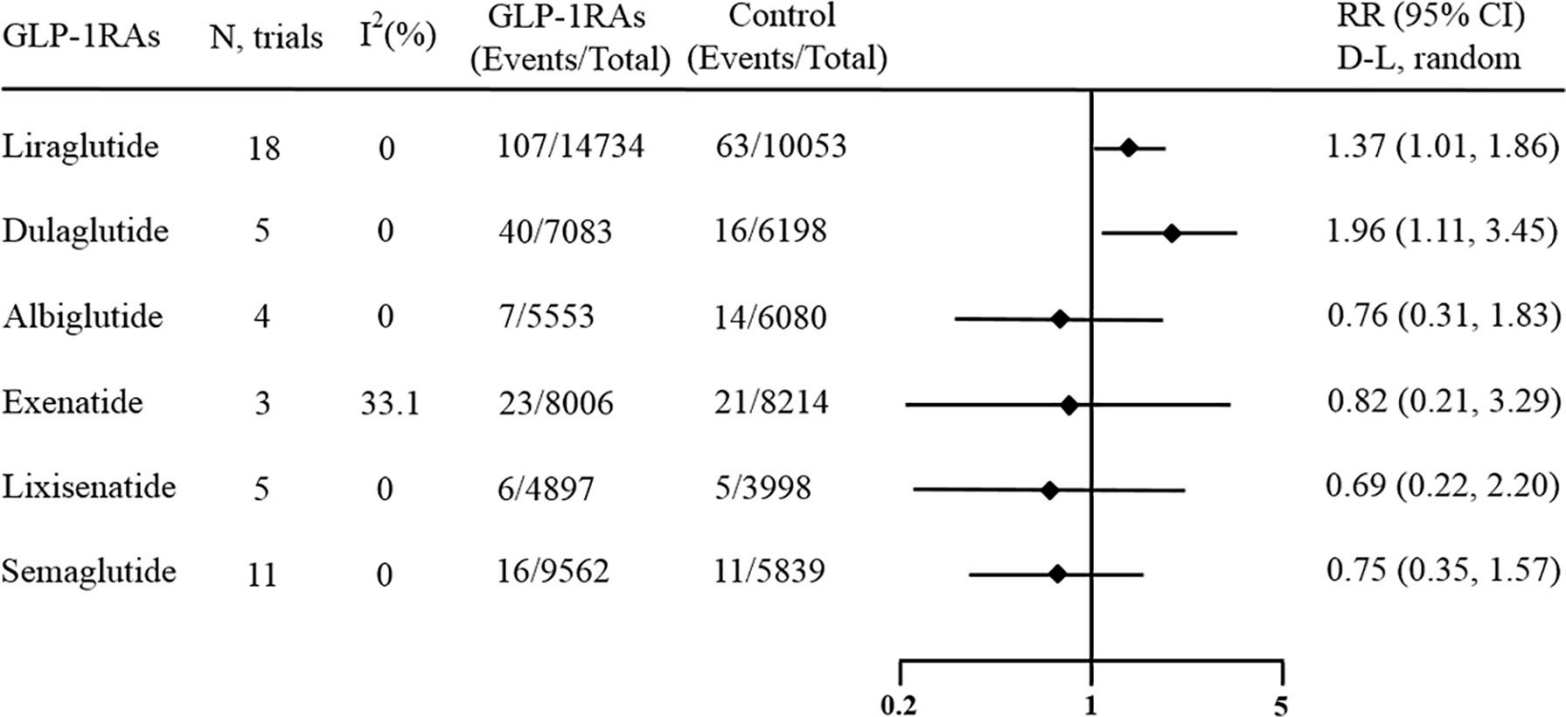
Forest plot of specific GLP-1 receptor agonists versus comparators on risk of overall thyroid disorders. GLP-1RAs, GLP-1 receptor agonists; RR, risk ratios; CI, confidence interval.

Among all 45 enrolled trials, 18 trials including 24787 patients used liraglutide as the experimental agent. Compared with placebo or other interventions, treatment with liraglutide increased the risk of overall thyroid disorders by 37% (RR 1.37, 95% CI 1.01-1.86, p = 0.044; **Figure 3**), and no statistically significant between-study heterogeneity was observed (I^2^ = 0.0%, p = 0.933).

Moreover, another 5 trials including 13281 patients provided information about the risk of thyroid disorders in patients treated with dulaglutide. This result showed that compared with placebo or other interventions, dulaglutide significantly increased the incidence of overall thyroid disorders by 96% (RR 1.96, 95% CI 1.11-3.45, p = 0.020; **Figure 3**), and no statistically significant between-study heterogeneity was observed (I^2^ = 0.0%, p = 0.965).

However, no effect against overall thyroid disorders was found for other GLP-1 receptor agonists. There were 11 studies including 15401 patients that regarded semaglutide as the experimental agent, and the pooled RR of overall thyroid disorders in patients receiving semaglutide versus other interventions was 0.75 (95% CI 0.35‐1.57; **Figure 3**). Whether oral semaglutide or subcutaneous semaglutide, the results showed that they had no significant effects on the occurrence of overall thyroid disorders (**Figure S9** and **Figure S10**). There were 5 studies including 8895 patients that regarded lixisenatide as the experimental agent, and the pooled RR of overall thyroid disorders in patients receiving lixisenatide versus other interventions was 0.69 (95% CI 0.22‐2.20; **Figure 3**). There were 3 studies including 16220 patients that regarded exenatide as the experimental agent, and the pooled RR of overall thyroid disorders in patients receiving exenatide versus other interventions was 0.82 (95% CI 0.21‐3.29; **Figure 3**). There were 3 studies including 11633 patients that regarded albiglutide as the experimental agent, and the pooled RR of overall thyroid disorders in patients receiving albiglutide versus other interventions was 0.76 (95% CI 0.31‐1.83; **Figure 3**). Most of the above meta-analyses had no heterogeneity (I^2^ = 0%), while one had medium heterogeneity (I^2^ = 33.1%).

### Subgroup Analyses and Meta-Regression Analyses

**Figure 4:**
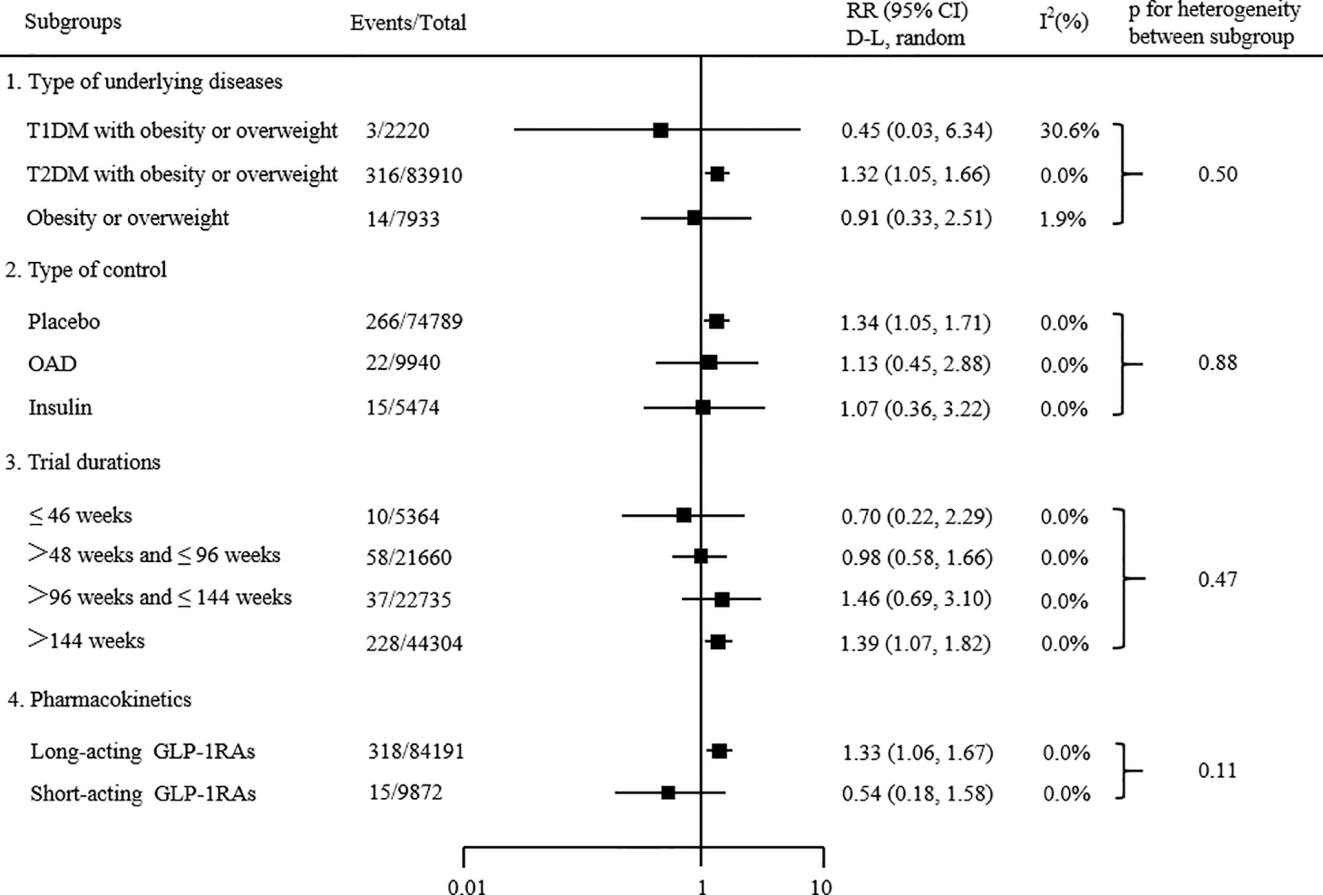
Subgroup analyses of the effects of GLP-1 receptor agonists on the risk of overall thyroid disorders. P value calculated by χ^2^ statistics is shown. Statistical significance of results from meta-regression was consistent.

Subgroup analyses based on type of underlying diseases, type of control, trial durations and pharmacokinetics. The results showed that the type of underlying diseases, type of control, trial durations and pharmacokinetics did not significantly affect the effects of GLP-1 receptor agonists on overall thyroid disorders (all P _subgroup _> 0.05; **Figure 4**). The statistical significance of the results from the meta-regression was consistent with the subgroup analyses.

## Discussion

This meta-analysis is the first large sample study that was designed to assess the relationship between the use of GLP-1 receptor agonists and the occurrence of various thyroid disorders. As a result, the following two major findings were produced. First, compared with placebo or other interventions, GLP-1 receptor agonists significantly increased the risk of overall thyroid disorders by 28%. Second, among GLP-1 receptor agonists, only liraglutide and dulaglutide showed increased trends in the risks of overall thyroid disorders compared with placebo and other antidiabetic drugs.

Despite the lack of consistent clinical and epidemiological evidence, the potential link between GLP-1 receptor agonists and thyroid cancer has received considerable attention. Rodent studies have shown that treatment with liraglutide or once-weekly exenatide is associated with thyroid C-cell proliferation and the formation of thyroid C-cell tumors (11, 63).

Therefore, the US Food and Drug Administration (FDA) prohibits these therapies for patients with an individual or family history of medullary thyroid carcinoma (MTC) or patients with multiple endocrine neoplasia syndrome type 2 (MEN2). However, these concerns are controversial in clinical trials. A retrospective analysis of the FDA’s AERS database found that the incidence of thyroid cancer treated with exenatide was 4.7 times that of the control drug (14). Similarly, analysis of data from the EudraVigilance database has found evidence from spontaneous reports that GLP-1 analogues are related to thyroid cancer in diabetic patients (64). However, a meta-analysis involving 25 studies showed that liraglutide had no significant correlation with the increased risk of thyroid cancer (65). Although our meta-analysis also showed that GLP-1 receptor agonists did not increase the risk of thyroid cancer compared to placebo or other interventions, in combination with previously available evidence, patients at risk for thyroid cancer should be prescribed GLP-1 receptor agonists with caution.

To date, the potential mechanism of the unfavorable effects of GLP-1 receptor agonists on thyroid disorders has not been completely clear. The possible mechanisms are as follows. First, it was reported that the mechanism of C-cell transformation in rodents is by activation of the GLP-1 receptor on the C cell, and a study has shown that GLP-1 receptor stimulation is a better predictor of C-cell hyperplasia than plasma drug concentrations of exenatide and liraglutide (66, 67). Second, in addition to medullary thyroid carcinoma and C-cell hyperplasia, the expression of GLP-1 receptors in papillary thyroid carcinoma (PTC) has been demonstrated. Gier et al. (68) reported positive immunoreactivity for GLP-1 receptors in PTC tissues, detected using a polyclonal anti-GLP-1 receptors antibody. Meanwhile, they reported that GLP-1 receptors were expressed differently in non-neoplastic thyroid tissues according to different inflammatory states. GLP-1 receptors were expressed in normal thyroid tissues with inflammation, but not in normal thyroid tissues without inflammation. In addition, another study also confirmed the expression of GLP-1 receptors in PTC and the expression rate of GLP-1 receptors in PTC, which was almost 30% (69). Korner et al. (70) ascertained the expression of GLP-1 receptors in various human thyroid tissues by scintigraphy and demonstrated that few normal thyroid tissue expressed GLP-1 receptors. Therefore, GLP-1 receptors may be abnormally induced in cells derived from thyroid follicles through inflammation, cell proliferation or tumorigenesis. However, some of the mentioned studies used GLP-1 receptor antibodies lacking specificity (71, 72). Using another detection method, Waser et al. found that neither normal nor hyperplastic human thyroids containing parafollicular C cells express GLP-1 receptors (73). At present, the presence and importance of GLP-1 receptors in normal human thyroid remains controversial. Third, GLP-1 might work through the phosphoinositol-3 kinase/AKT serine/threonine kinase (PI3K/Akt) pathway and/or mitogen-activated protein kinase/extracellular signal-regulated kinase (MAPK/Erk) pathway. These two signaling pathways are also critical in regulating cell growth and proliferation; accordingly, they are closely related to cancer, including PTC. These two signaling pathways are significant pathways for regulating cell growth and proliferation, and thus they are closely related to cancer formation (74). Finally, the GlP-1 receptor may be associated with triiodothyronine (T3) levels. GLP-1 stimulates type 3 iodothyronine deiodinase (D3) expression through the GLP-1 receptor, and the regulation of intracellular (T3) concentration by D3 may be involved in the stimulation of insulin secretion by GLP-1 (75). In addition, a clinical study showed that exenatide treatment for 6 months significantly reduced the serum TSH concentration in diabetic patients without thyroid disease (76). In conclusion, some animal studies have provided evidence that the use of GLP-1 receptor agonists increases the risk of thyroid disease, but this evidence has not been confirmed in humans. Therefore, we performed this meta-analysis to clarify the association of GLP-1 receptor agonists with thyroid disease in clinical studies and preparation for future studies in humans. Further prospective studies should be carried out to determine the potential effects of GLP-1 receptor agonists on thyroid disease.

In the analysis of different types of GLP-1 receptor agonists, we found that liraglutide and dulaglutide were significantly associated with an increased risk of overall thyroid disorders. However, individual tolerability and safety to GLP-1RA may vary due to differences in molecular structures (77). Furthermore, these different findings could explain with an imbalanced sample size. It is worth noting that the significantly increased risk of liraglutide is largely driven by the LEADER trial (20) and that of dulaglutide is largely driven by the REWIND test (42), both of which contributed more than 75% of the weight to the overall results. Due to the lack of sufficient research, we cannot draw a decisive conclusion until further research provides more information. Among the included studies, only one was related to short-acting exenatide (39), and two were long-acting exenatide (18, 40). Due to the small number of studies, we did not separately analyze according to pharmacokinetics.

This review has two main strengths. First, this is the first meta-analysis to comprehensively assess the risks of various thyroid diseases associated with the use of GLP-1 receptor agonists. Moreover, all included studies were RCTs. Second, no or only mild heterogeneity was found in any of the meta-analyses conducted in the present study.

We acknowledge that our study has several limitations. First, almost every included study did not consider thyroid events as the main result, only regarded them as safety results and did not monitor the changes in thyroid function at the same time. In addition, only trials reporting thyroid events were included in this analysis, leading to an unclear risk of reporting bias. Second, although this analysis included 45 studies with a fairly large sample size, the low incidence of thyroid events resulted in a wide confidence interval that reduced the certainty of our findings. Moreover, the study groups considerably differ in size (52600 vs. 41463). Considering the slight difference in the rate of thyroid disorders (0.39 vs. 0.31%), a significant influence on the primary endpoint cannot be ruled out. The third limitation is that there may be the potential for numerous indirect effects or confounding. For example, reduction in BMI in obesity patients, caloric restriction, and illness are all associated with different thyroid function test (TFT) changes. Patients may be more stringently screened, particularly for thyroid nodules/cancer in patients receiving GLP-1 receptor agonists. Another limitation is that for thyroid cancer, reporting specifically the cases of MTC vs. PTC would further the goal of elucidating mechanisms of thyroid disease. However, we found that some studies did not specify the type of thyroid cancer, which would affect the accuracy of the results. Due to the lack of standardization of adverse event reports and original data, we cannot make comparisons according to different types. Finally, although our meta-analysis showed that GLP-1 receptor agonists increased the risk of overall thyroid disorder, due to the decrease in sample size, it did not show statistically significant results for specific thyroid disorder. Future large long-term RCTs with primary or secondary outcomes, including thyroid disorders and real-world data, are needed to elucidate the association between GLP-1 receptor agonists and the risk of various thyroid disorders, particularly thyroid cancer.

## Conclusion

In conclusion, compared with placebo or other interventions, GLP-1 receptor agonists did not increase or decrease the risk of thyroid cancer, hyperthyroidism, hypothyroidism, thyroiditis, thyroid mass and goiter. Due to the low incidence of various thyroid disorders, these findings still need to be verified by further studies.

## Data Availability Statement

The datasets presented in this study can be found in online repositories. The names of the repository/repositories and accession number(s) can be found in the article/**Supplementary Material**.

## Author Contributions

JL and XL designed and outlined the work; WH, RS, RC, CL, RG, WT, JZ and QZ drafted and revised the manuscript. Both authors approved the final version of the article and agree to be accountable for all aspects of the work. All authors contributed to the article and approved the submitted version.

## Funding

This work was supported in part by National Natural Science Foundation of China (No. 82000799), Research Project Supported by Shanxi Scholarship Council of China (No. 2020-187), Scientific Research Project of Shanxi Provincial Health Committee (No.2021068), The Doctoral Foundation of the Second Hospital of Shanxi Medical University (No. 20200112) and Natural Science Foundation of Shanxi Province (No. 202103021224243).

## Conflict of Interest

The authors declare that the research was conducted in the absence of any commercial or financial relationships that could be construed as a potential conflict of interest.

## Publisher’s Note

All claims expressed in this article are solely those of the authors and do not necessarily represent those of their affiliated organizations, or those of the publisher, the editors and the reviewers. Any product that may be evaluated in this article, or claim that may be made by its manufacturer, is not guaranteed or endorsed by the publisher.
