# Supplementary material for: Use of GLP-1 Receptor Agonists and Occurrence of Thyroid Disorders: a Meta-Analysis of Randomized Controlled Trials
Source: Front Endocrinol (Lausanne). 2022 Jul 11;13:927859. doi: 10.3389/fendo.2022.927859 (PMC9309474; doi:10.3389/fendo.2022.927859)
Supplement: Supplementary file 1 [file DataSheet_1.pdf]

Appendix

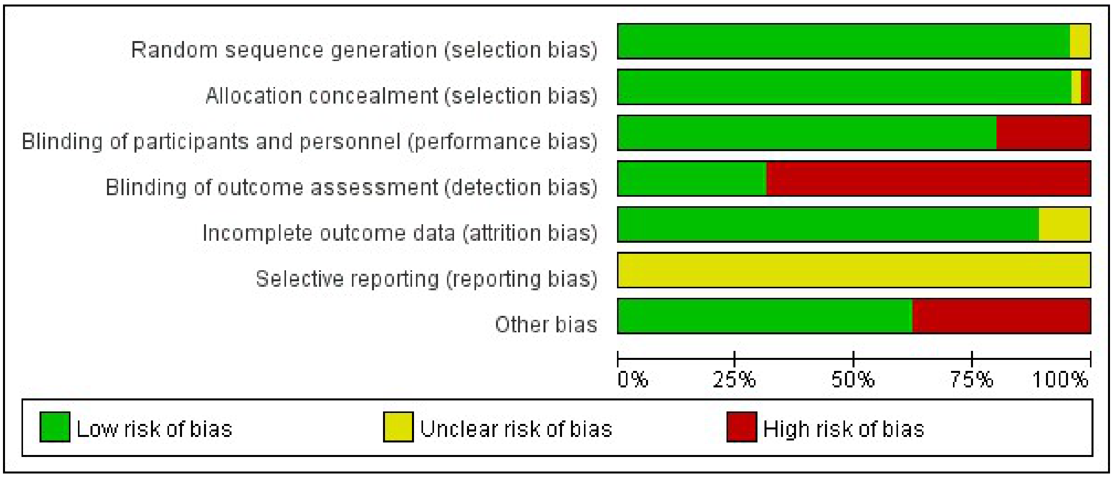

Figure S1. Quality assessment (Cochrane risk of bias tool) for included RCTs.

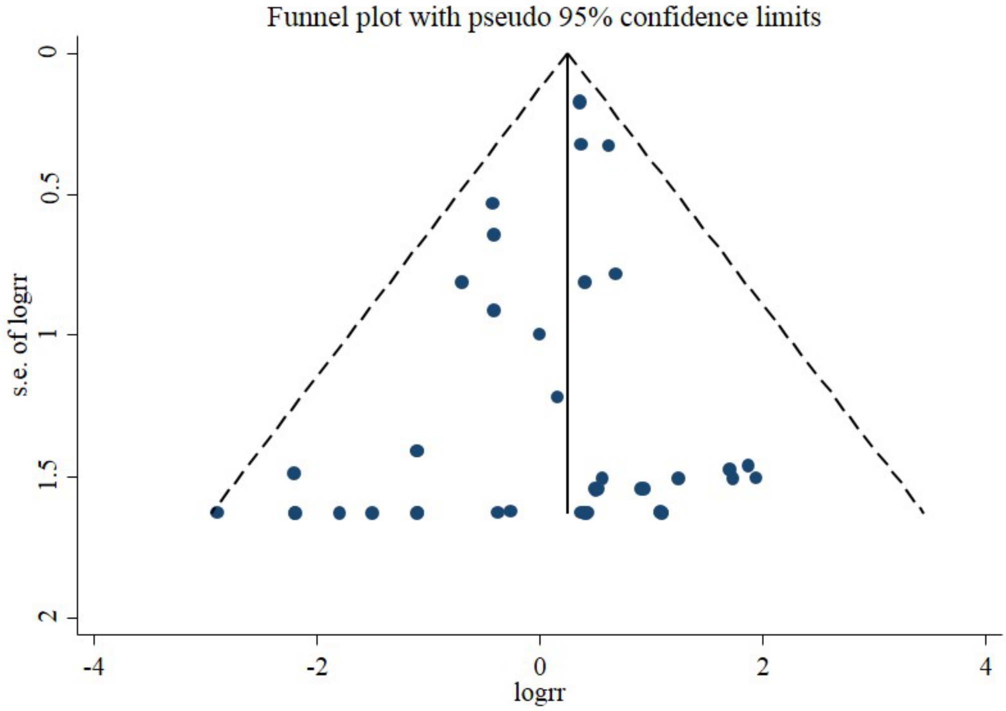

Figure S2. Funnel plot for the comparison of the incidence of overall thyroid disorders with the use of GLP-1 receptor agonists versus placebo or other antidiabetic treatments.

Thyroid Cancer	GLP-1RAs (Events/Total)	Control (Events/Total)	RR (95% CI) D-L, random	Weight (%)
Unger et al 2021	1/996	0/995	3.00 (0.12, 73.48)	1.67
Wadden et al 2021	1/407	0/204	1.51 (0.06, 36.84)	1.68
Wilding et al 2021	1/1306	0/655	1.51 (0.06, 36.91)	1.67
Gerstein et al 2021	2/2717	0/1359	2.50 (0.12, 52.08)	1.86
Wadden et al 2020	1/142	0/140	2.96 (0.12, 72.00)	1.68
Yamada et al 2020	1/194	0/49	0.77 (0.03, 18.60)	1.69
Gerstein et al 2019	8/4949	3/4952	2.67 (0.71, 10.05)	9.74
Wang et al 2019	1/505	0/250	1.49 (0.06, 36.40)	1.68
Aroda et al 2019	1/525	0/178	1.02 (0.04, 24.95)	1.68
Pratley et al 2019	2/569	0/142	1.25 (0.06, 25.98)	1.86
Husain et al 2019	2/1591	0/1592	5.00 (0.24, 104.13)	1.86
O'Neil et al 2018	0/821	1/136	0.06 (0.00, 1.36)	1.68
le et al 2017	3/1505	0/749	3.49 (0.18, 67.40)	1.95
Holman et al 2017	12/7356	6/7396	2.01 (0.76, 5.35)	17.85
Ahrén et al 2017	1/818	0/407	1.49 (0.06, 36.61)	1.67
Aroda et al 2017	0/722	1/360	0.17 (0.01, 4.08)	1.67
Zang et al 2016	0/183	1/184	0.34 (0.01, 8.17)	1.68
Mathieu et al 2016	0/1042	1/347	0.11 (0.00, 2.72)	1.67
Marso et al 2016	7/4668	5/4672	1.40 (0.45, 4.41)	13.02
Marso et al 2016	1/1648	3/1649	0.33 (0.03, 3.20)	3.35
Pi-Sunyer et al 2015	1/959	0/487	1.52 (0.06, 37.37)	1.67
Davies et al 2015	0/634	1/212	0.11 (0.00, 2.73)	1.68
Home et al 2015	0/271	1/392	0.48 (0.02, 11.78)	1.68
Giorgino et al 2015	1/545	0/262	1.45 (0.06, 35.35)	1.68
Weinstock et al 2015	2/606	0/492	4.06 (0.20, 84.39)	1.86
Pfeffer et al 2015	1/3034	1/3034	1.00 (0.06, 15.98)	2.23
Ahrén et al 2014	1/302	2/710	1.18 (0.11, 12.91)	2.98
Gough et al 2014	1/1247	0/413	1.00 (0.04, 24.38)	1.67
Wadden et al 2013	3/212	0/210	6.93 (0.36, 133.43)	1.96
Ahrén et al 2013	1/510	0/170	1.00 (0.04, 24.53)	1.68
Gallwitz et al 2012	0/490	1/487	0.33 (0.01, 8.11)	1.67
Bergenstal et al 2010	0/160	1/331	0.69 (0.03, 16.78)	1.68
Seino et al 2010	1/268	0/132	1.48 (0.06, 36.17)	1.68
Nauck et al 2009	1/724	1/363	0.50 (0.03, 7.99)	2.23
Garber et al 2009	5/498	0/248	5.49 (0.30, 98.87)	2.05
**Overall**	**63/43124**	**29/34359**	**1.30 (0.86, 1.97)**	**100.00**

Heterogeneity: $I^2 = 0\%$, $p = 0.97$
Test for overall effect: $p = 0.21$

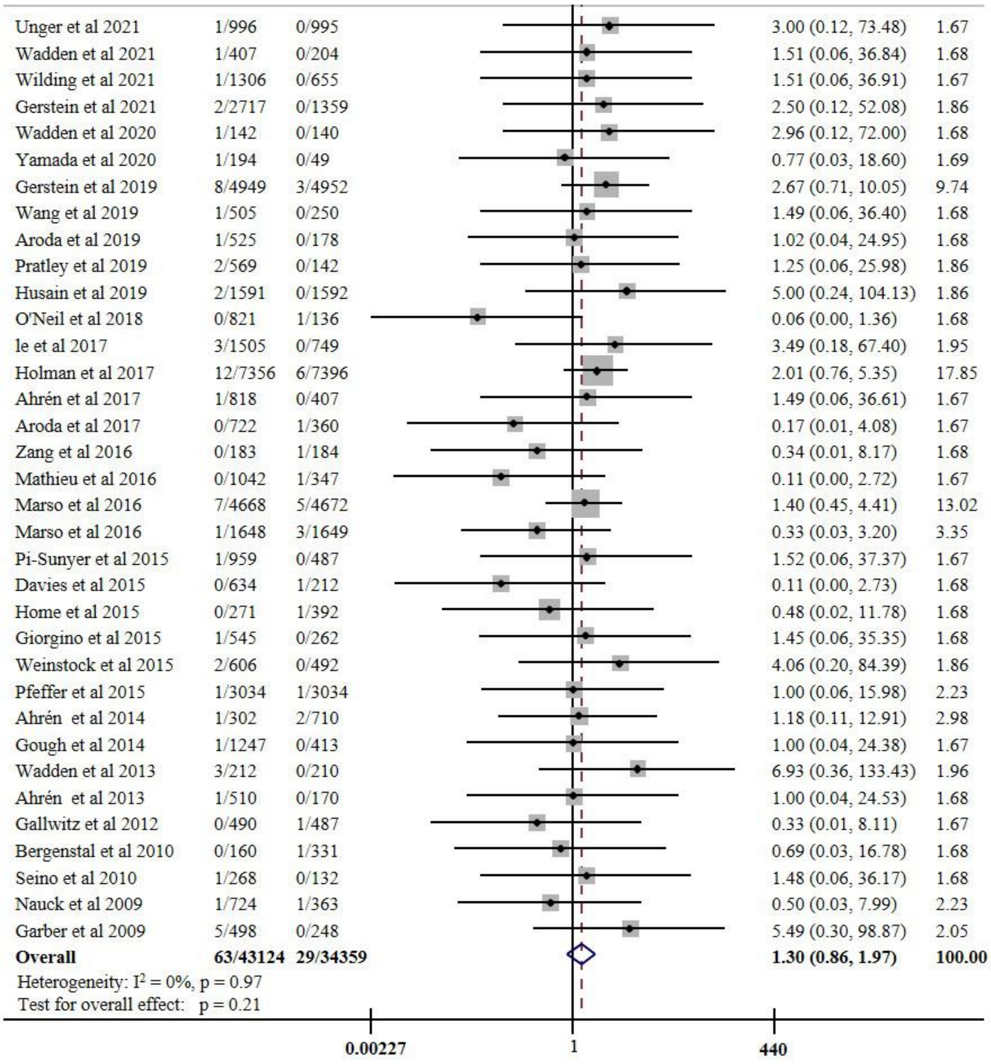

Figure S3. Forest plot of GLP-1 receptor agonists versus comparators on risk of thyroid cancer. GLP-1RAs: GLP-1 receptor agonists, RR: risk ratios, CI: confidence interval

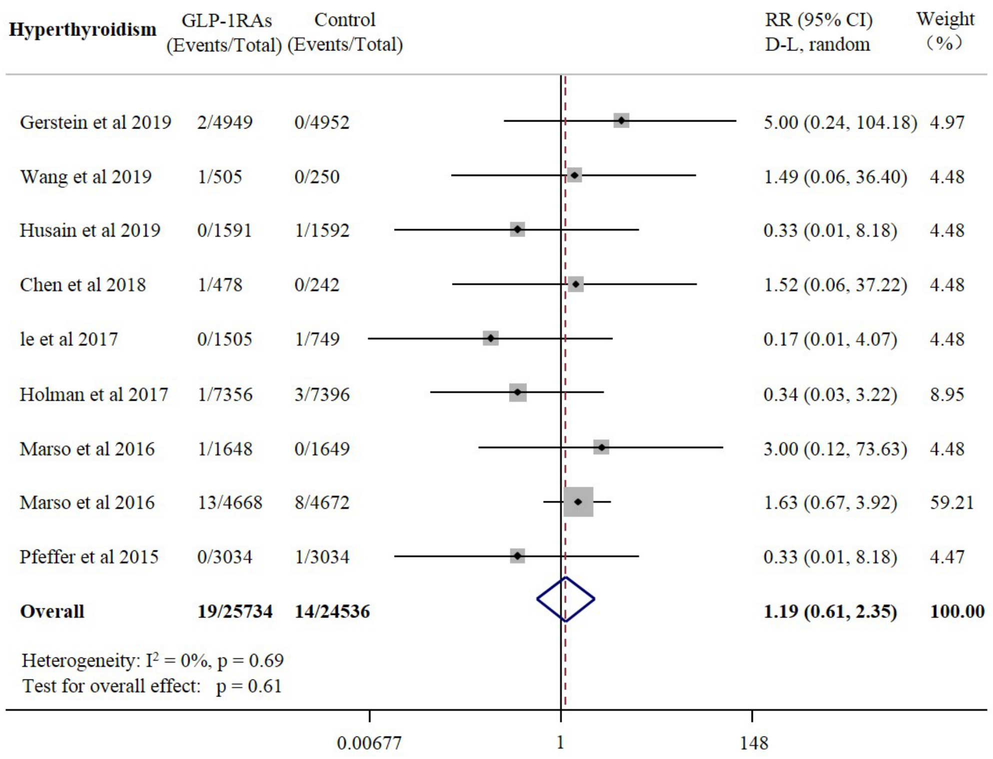

Figure S4. Forest plot of GLP-1 receptor agonists versus comparators on risk of hyperthyroidism. GLP-1RAs: GLP-1 receptor agonists, RR: risk ratios, CI: confidence interval

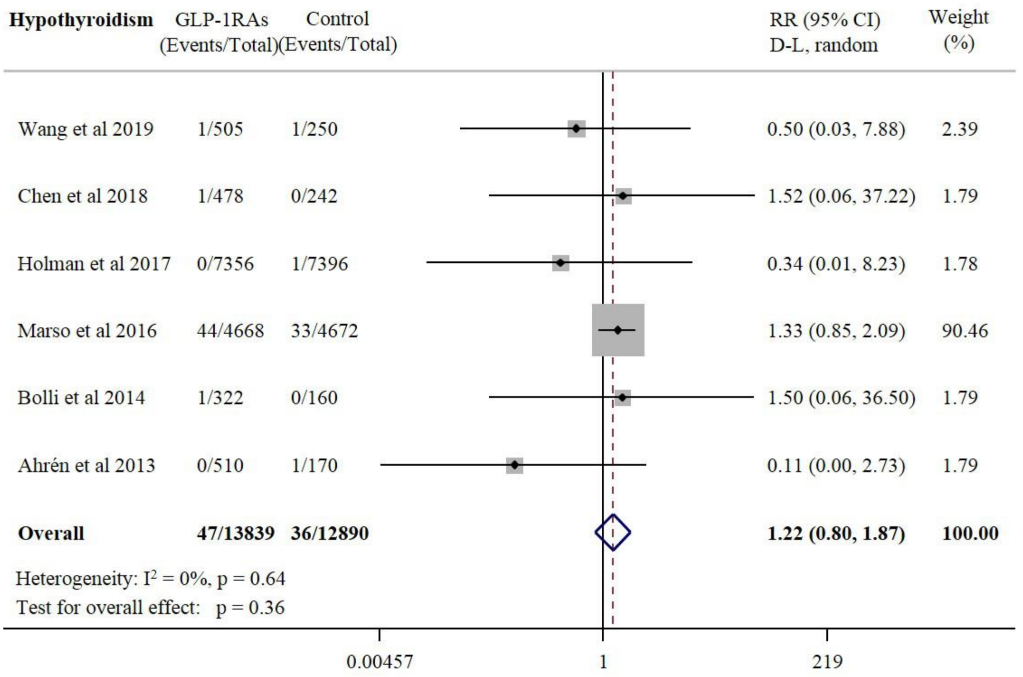

Figure S5. Forest plot of GLP-1 receptor agonists versus comparators on risk of hypothyroidism. GLP-1RAs: GLP-1 receptor agonists, RR: risk ratios, CI: confidence interval

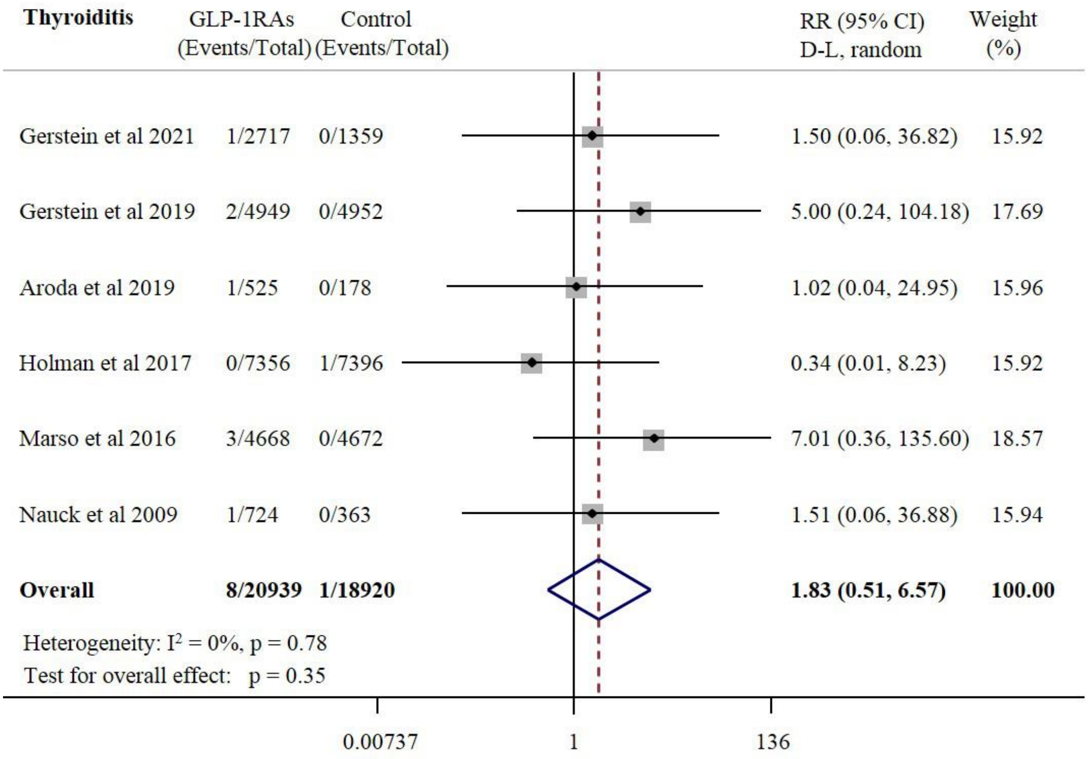

Figure S6. Forest plot of GLP-1 receptor agonists versus comparators on risk of thyroiditis. GLP-1RAs: GLP-1 receptor agonists, RR: risk ratios, CI: confidence interval

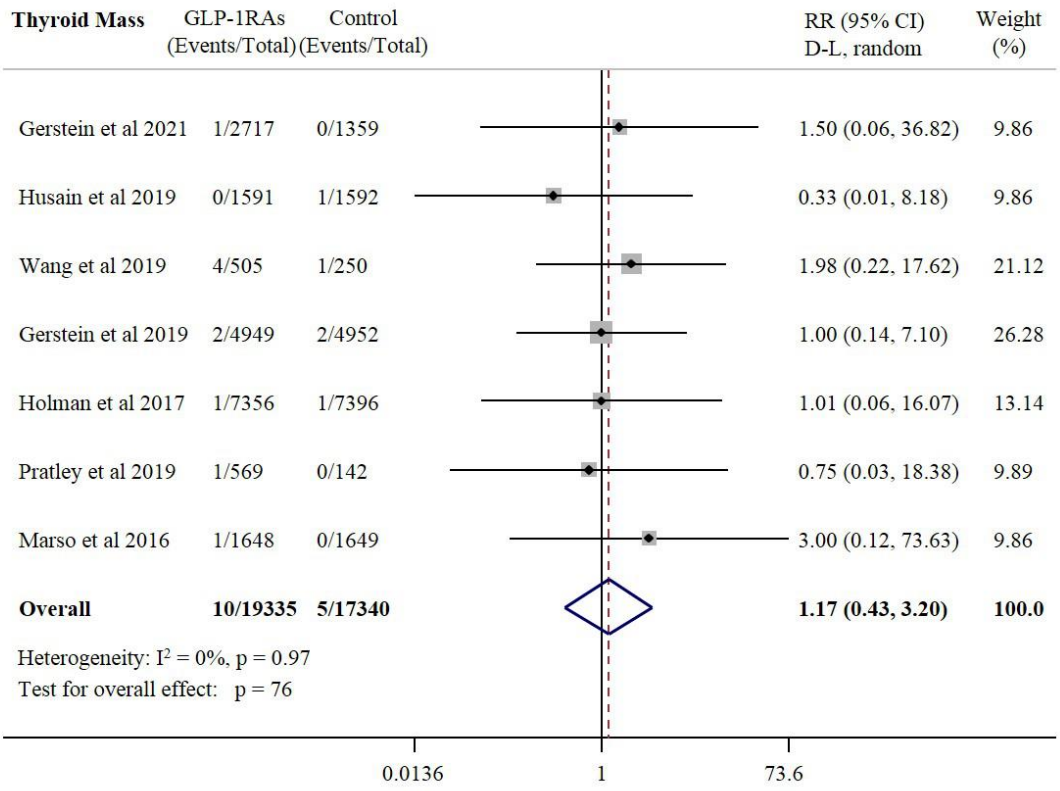

Figure S7. Forest plot of GLP-1 receptor agonists versus comparators on risk of thyroid mass. GLP-1RAs: GLP-1 receptor agonists, RR: risk ratios, CI: confidence interval

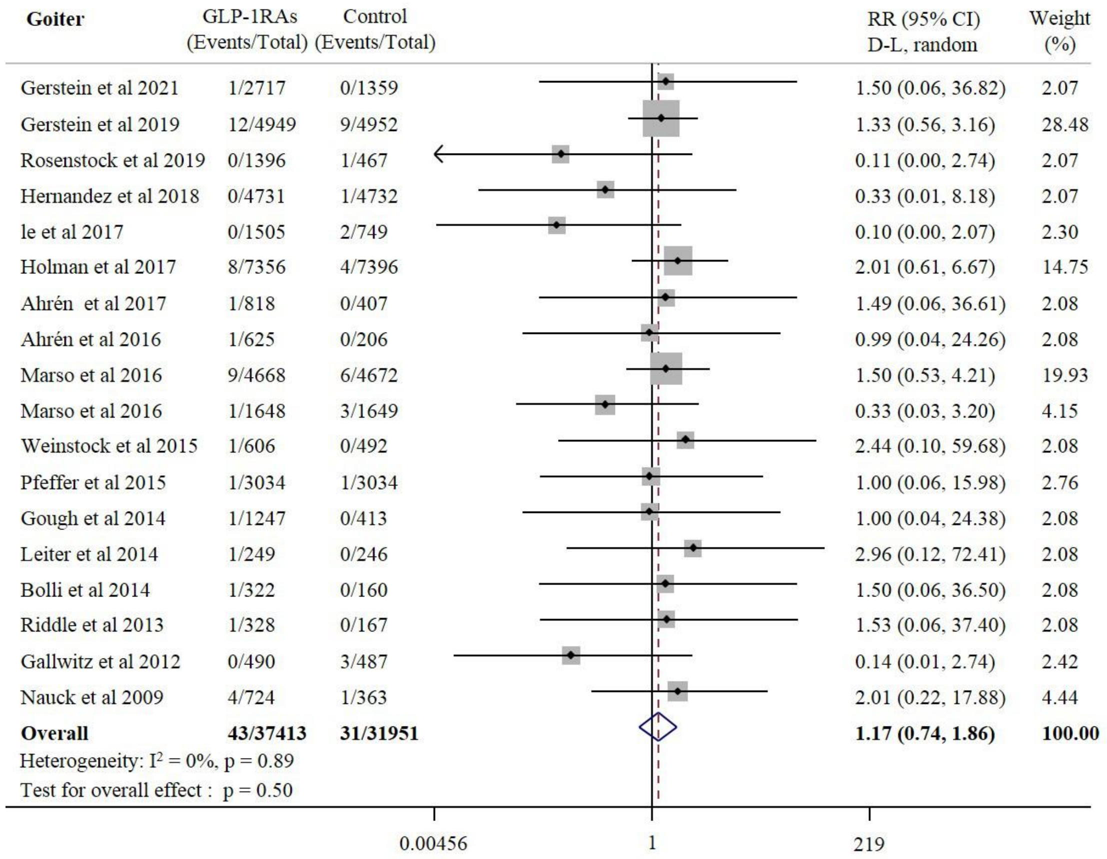

Figure S8. Forest plot of GLP-1 receptor agonists versus comparators on risk of goiter. GLP-1RAs: GLP-1 receptor agonists, RR: risk ratios, CI: confidence interval

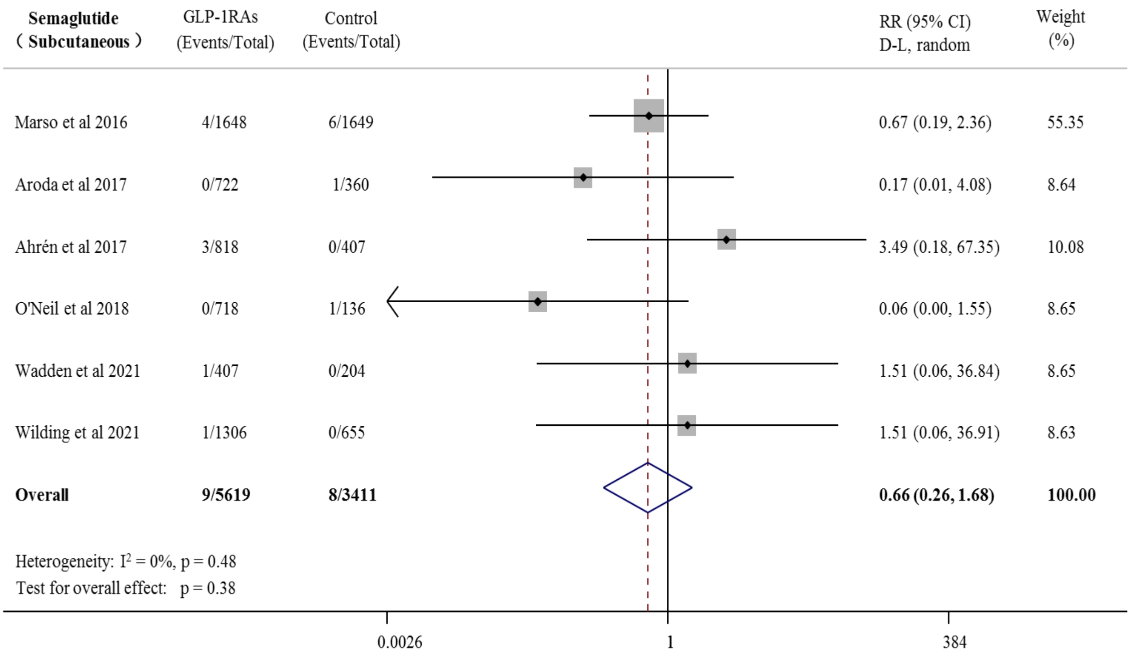

Figure S9. Forest plot of subcutaneous semaglutide versus comparators on risk of overall thyroid disorders. GLP-1RAs: GLP-1 receptor agonists, RR: risk ratios, CI: confidence interval

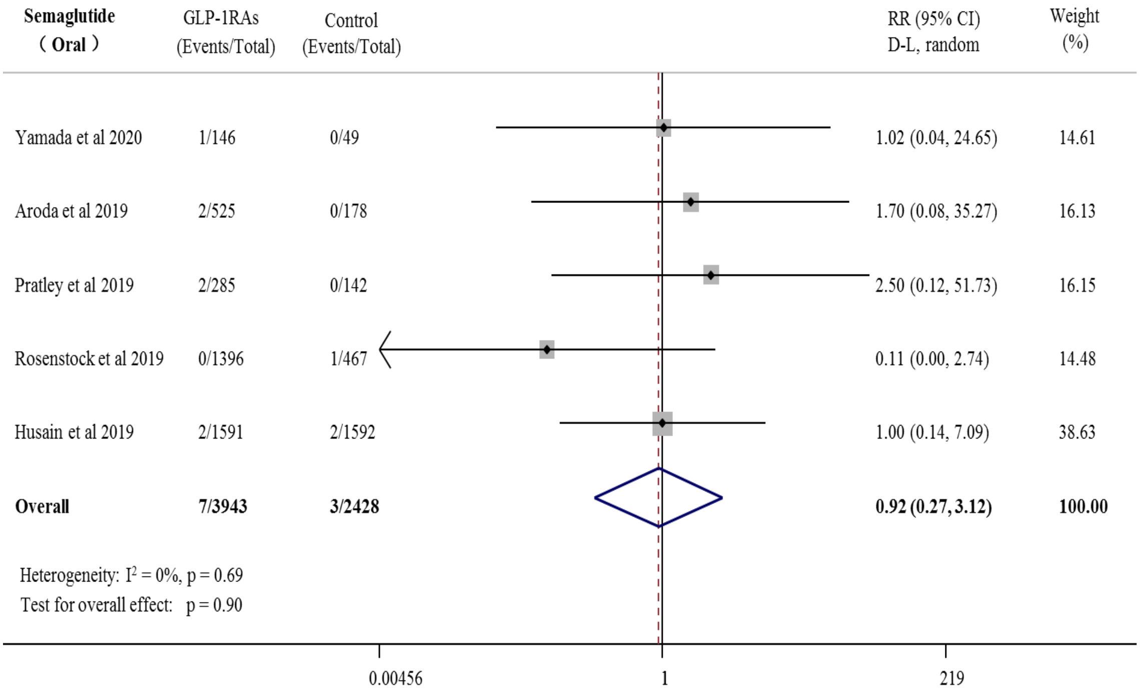

Figure S10. Forest plot of oral semaglutide versus comparators on risk of overall thyroid disorders. GLP-1RAs: GLP-1 receptor agonists, RR: risk ratios, CI: confidence interval

Table S1. Events of thyroid outcomes for included studies

Study	Thyroid Cancer [a]		Hyperthyroidism [a]		Hypothyroidism [a]		Thyroiditis [a]		Thyroid Mass [a]		Goiter [a]		Unspecified thyroid disorder	
	Experimental	Control	Experimental	Control	Experimental	Control	Experimental	Control	Experimental	Control	Experimental	Control	Experimental	Control
Unger et al 2022[20]	1[b]	0	0	0	0	0	0	0	0	0	0	0	0	0
Garveyet al 2020[21]	0	0	0	0	0	0	0	0	0	0	0	0	1[b]	0
Wadden et al 2020[22]	1[b]	0	0	0	0	0	0	0	0	0	0	0	0	0
le et al 2017[23]	3[b]	0	0	1[b]	0	0	0	0	0	0	0	2[b]	0	0
Pi-Sunyer et al 2015[24]	1[b]	0	0	0	0	0	0	0	0	0	0	0	0	0
Zang et al 2016[25]	0	1[b]	0	0	0	0	0	0	0	0	0	0	0	0
Ahrén et al 2016[26]	0	0	0	0	0	0	0	0	0	0	1[b]	0	1[b]	0
Mathieu et al 2016[27]	0	1[b]	0	0	0	0	0	0	0	0	0	0	0	0
Marso et al 2016[19]	7[b]	5[b]	13[b]	8[b]	44[b]	33[b]	3[b]	0	0	0	9[b]	6[b]	1[b]	2[b]
Davies et al 2015[28]	0	1[b]	0	0	0	0	0	0	0	0	0	0	1[b]	0
Gough et al 2014[29]	1[b]	0	0	0	0	0	0	0	0	0	1[b]	0	0	0
Wadden et al 2013[30]	3[b]	0	0	0	0	0	0	0	0	0	0	0	0	0
Seino et al 2010[31]	1[b]	0	0	0	0	0	0	0	0	0	0	0	0	0
Pratley et al 2010[32]	0	0	0	0	0	0	0	0	0	0	0	0	1[b]	0
Nauck et al 2009[33]	1[b]	1[b]	0	0	0	0	1[b]	0	0	0	4[b]	1[b]	0	0
Garber et al 2009[34]	5[b]	0	0	0	0	0	0	0	0	0	0	0	1[b]	0
Hernandez et al 2018[35]	0	0	0	0	0	0	0	0	0	0	0	1[c]	0	0
Home et al 2015[36]	0	1[b]	0	0	0	0	0	0	0	0	0	0	5[b]	10[b]
Ahrén et al 2014[37]	1[b]	2[b]	0	0	0	0	0	0	0	0	0	0	0	0
Leiter et al 2014[18]	0	0	0	0	0	0	0	0	0	0	1[b]	0	0	0
Holman et al 2017[17]	12[b]	6[b]	1[b]	3[b]	0	1[b]	0	1[b]	1[b]	1[b]	8[b]	4[b]	1[b]	0
Gallwitz et al 2012[38]	0	1[c]	0	0	0	0	0	0	0	0	0	3[c]	0	0
Bergenstal et al 2010[39]	0	1[c]	0	0	0	0	0	0	0	0	0	0	0	0
Wang et al 2019[40]	1[b]	0	1[b]	0	1[b]	1[b]	0	0	4[b]	1[b]	0	0	1[b]	0
Gerstein et al 2019[41]	8[b]	3[b]	2[b]	0	0	0	2[b]	0	2[b]	2[b]	12[b]	9[b]	0	0
Chen et al 2018[42]	0	0	1[b]	0	1[b]	0	0	0	0	0	0	0	0	0
Weinstock et al 2015[43]	2[b]	0	0	0	0	0	0	0	0	0	1[b]	0	0	0
Giorgino et al 2015[44]	1[b]	0	0	0	0	0	0	0	0	0	0	0	0	0
Rosenstock et al 2016[45]	0	0	0	0	0	0	0	0	0	0	0	0	0	1[b]
Pfeffer et al 2015[46]	1[b]	1[b]	0	1[b]	0	0	0	0	0	0	1[b]	1[b]	0	0
Bolli et al 2014[47]	0	0	0	0	1[c]	0	0	0	0	0	1[c]	0	0	0
Ahrén et al 2013[48]	1[c]	0	0	0	0	1[c]	0	0	0	0	0	0	0	0
Riddle et al 2013[49]	0	0	0	0	0	0	0	0	0	0	1[c]	0	0	0
Wilding et al 2021[50]	1[b]	0	0	0	0	0	0	0	0	0	0	0	0	0
Wadden et al 2021[51]	1[b]	0	0	0	0	0	0	0	0	0	0	0	0	0

Yamada et al 2020[52]	1 [b]	0	0	0	0	0	0	0	0	0	0	0	0	0
Husain et al 2019[53]	2 [b]	0	0	1 [b]	0	0	0	0	0	1 [b]	0	0	0	0
Rosenstock et al 2019[54]	0	0	0	0	0	0	0	0	0	0	0	1 [b]	0	0
Pratley et al 2019[55]	2 [b]	0	0	0	0	0	0	0	1 [b]	0	0	0	0	0
Aroda et al 2019[56]	1 [b]	0	0	0	0	0	1 [b]	0	0	0	0	0	0	0
O'Neil et al 2018[57]	0	1 [b]	0	0	0	0	0	0	0	0	0	0	0	0
Ahrén et al 2017[58]	1 [b]	0	0	0	0	0	0	0	0	0	1 [b]	0	1 [b]	0
Aroda et al 2017[59]	0	1 [b]	0	0	0	0	0	0	0	0	0	0	0	0
Marso et al 2016[16]	1 [b]	3 [b]	1 [b]	0	0	0	0	0	1 [b]	0	1 [b]	3 [b]	0	0
Gerstein et al 2021[60]	2 [b]	0	0	0	0	0	1 [b]	0	1 [b]	0	1 [b]	0	0	0

[a] Term from vocabulary, MedDRA

[b] Indicates events were collected by systematic assessment

[c] Indicates events were collected by non-systematic assessment

Table S2. Definitions of thyroid outcomes for included studies

Outcomes	Definition
Thyroid cancer	According to the MedDRA dictionary, all the events defined as ‘Papillary thyroid cancer, Medullary Thyroid cancer, Thyroid cancer, Thyroid neoplasm, Benign neoplasm of thyroid gland, Thyroid carcinoma’ were considered.
Hyperthyroidism	According to the MedDRA dictionary, all the events defined as ‘Hyperthyroidism’ were considered.
Hypothyroidism	According to the MedDRA dictionary, all the events defined as ‘Hypothyroidism’ were considered.
Thyroiditis	According to the MedDRA dictionary, all the events defined as ‘Thyroiditis, Autoimmune thyroiditis, Thyroiditis subacute’ were considered.
Thyroid mass	According to the MedDRA dictionary, all the events defined as ‘Thyroid mass’ were considered.
Goiter	According to the MedDRA dictionary, all the events defined as ‘Goitre or Toxic nodular goitre’ were considered.
Unspecified thyroid disorder	Some thyroid disorders did not specify in the references or ClinicalTrials.gov.
